# Detection of COVID-19 in Chest X-ray Images: A Big Data Enabled Deep Learning Approach

**DOI:** 10.3390/ijerph181910147

**Published:** 2021-09-27

**Authors:** Mazhar Javed Awan, Muhammad Haseeb Bilal, Awais Yasin, Haitham Nobanee, Nabeel Sabir Khan, Azlan Mohd Zain

**Affiliations:** 1 Department of Software Engineering, University of Management and Technology, Lahore 54770, Pakistan; s2020114006@umt.edu.pk; 2 Department of Computer Engineering, National University of Technology, Islamabad 44000, Pakistan; awaisyasin@nutech.edu.pk; 3 College of Business, Abu Dhabi University, Abu Dhabi 59911, United Arab Emirates; 4 Oxford Centre for Islamic Studies, University of Oxford, Marston Rd, Headington, Oxford OX3 0EE, UK; 5 Faculty of Humanities & Social Sciences, University of Liverpool, 12 Abercromby Square, Liverpool L69 7WZ, UK; 6 Department of Computer Science, University of Management and Technology, Lahore 54770, Pakistan; nabeel.bloch@umt.edu.pk; 7 UTM Big Data Centre, School of Computing, Universiti Teknologi Malaysia, Skudai 81310, Malaysia; azlanmz@utm.my

**Keywords:** COVID-19, corona virus, pneumonia, chest X-ray, CNN, transfer learning, big data, public health, data bricks, Apache Spark, ResNet50, InceptionV3, VGG19, SparkDL, machine learning, deep learning

## Abstract

Coronavirus disease (COVID-19) spreads from one person to another rapidly. A recently discovered coronavirus causes it. COVID-19 has proven to be challenging to detect and cure at an early stage all over the world. Patients showing symptoms of COVID-19 are resulting in hospitals becoming overcrowded, which is becoming a significant challenge. Deep learning’s contribution to big data medical research has been enormously beneficial, offering new avenues and possibilities for illness diagnosis techniques. To counteract the COVID-19 outbreak, researchers must create a classifier distinguishing between positive and negative corona-positive X-ray pictures. In this paper, the Apache Spark system has been utilized as an extensive data framework and applied a Deep Transfer Learning (DTL) method using Convolutional Neural Network (CNN) three architectures —InceptionV3, ResNet50, and VGG19—on COVID-19 chest X-ray images. The three models are evaluated in two classes, COVID-19 and normal X-ray images, with 100 percent accuracy. But in COVID/Normal/pneumonia, detection accuracy was 97 percent for the inceptionV3 model, 98.55 percent for the ResNet50 Model, and 98.55 percent for the VGG19 model, respectively.

## 1. Introduction

The primary instance of COVID-19 was accounted for in Wuhan, China [1]. The virus marked a pandemic on 11 March 2020 [2]. On 14 September 2021, the WHO recorded 4,636,153 deaths and 225,024,781 affirmed cases. As of 12 September 2021, an aggregate of 5,534,977,637 vaccine doses had been distributed [3,4]. This pandemic affected almost all countries.

There is pressure on testing laboratories due to the global epidemiological condition. COVID-19 tests are accessible to check for present or past diseases. A viral test decides if you are contaminated right now. Antigen testing and nucleic acid amplification tests (NAATs) are two types of viral tests that can be utilized. Neutralizer testing ought not to be used to analyze contamination that is present [5]. 

A chest X-ray is the most widely recognized imaging procedure used to analyze SARS-CoV-2 infection. Utilizing a Convolutional Neural Network to perceive COVID-19 radiology images gives extensive results, as per a couple of studies [6,7,8], and it merits further attention and significance.

The blend of the two innovations Apache Spark and Transfer Learning, permits information investigators to recognize photographs rapidly and effectively and encourages models that can run on clusters [9,10].

Big data analytics could be helpful for researchers as well as businesses. Its techniques have been implemented for fake news prevention, as mentioned in [11]. Similarly, in [12], social media are analyzed through big data and are used for stock-market predictions. It also has been used for review processing [13]. Moreover, in [14], the big data approach is used for sales analytics, such as predicting black Friday sales based on historical data.

**Figure 1:**
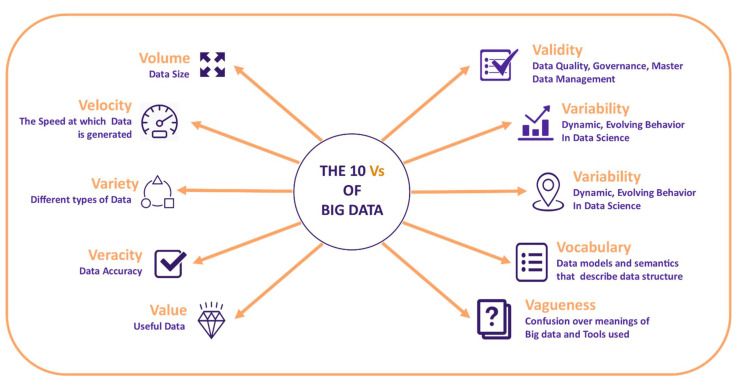
10’Vs of big data characteristics.

“Big Data” is an all-encompassing label that encompasses non-traditional tactics and innovation for acquiring, sorting, and producing experiences from massive datasets [15]. The volume of information has expanded drastically lately, requiring the improvement of data analytics frameworks. The Apache Spark framework [16] is the most notable stage for big data analytics. Spark can process information utilizing an assortment of structures. It is lightning quick, upholds various programming languages, incorporates machine-learning usefulness, and associates with multiple platforms [17]. It has characteristics described as 10 V’s in Figure 1.

Big data in medical care is developing to bring understanding from substantial information sets, and it is yielding tremendous results while bringing down costs and guaranteeing great patient care [18]. Apache Spark can assume an essential part in medical services investigation, helping clinicians better diagnose problems, especially picture classification [19]. Information-retrieval models need increasing detail as mentioned in [20], and information-extraction techniques were mentioned in [21].

Big data analytics is the practice of utilizing advanced analytical techniques to examine massive volumes of information. Scientists, organizations, and examiners can use extensive data analysis to settle on better and quicker choices.

They can utilize modern analytics procedures, such as machine learning, deep learning, prescient examination, and others to create novel thoughts, approaches, and experiences regarding big data [22]. The goal is to uncover hidden patterns and associations that disclose crucial information about the clients who developed it ahead of time. Big data analytics can improve findings and therapies, enhance administration quality at a lower cost, and deliver better outcomes [23].

**Figure 2:**
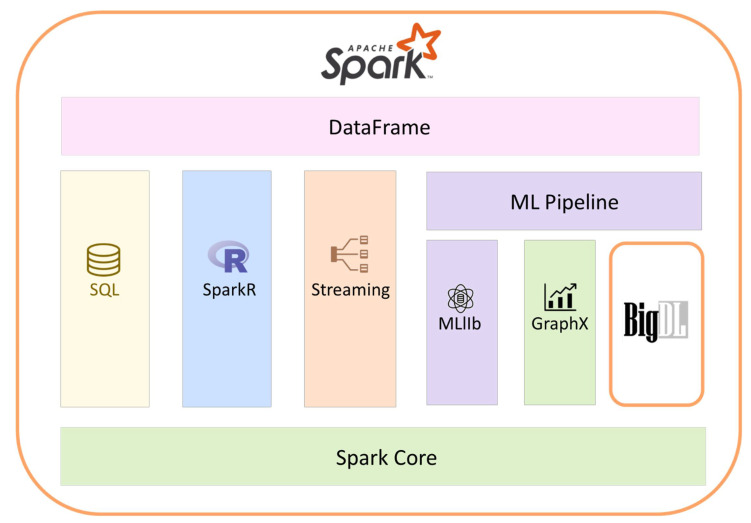
Apache Spark and its relation to BigDL library for deep learning.

Apache Spark is an open-source information handling system that spotlights speed, effortlessness, and progressed investigation. Spark runs in-memory on clusters, is not constrained by Hadoop’s two-stage MapReduce model, and is extremely fast [24]. Spark may run on its own, on top of tools, and it can read data directly from the file systems of these tools. Spark can deal with spilling notwithstanding in-memory preparing, diagram handling, and machine learning [25]. The Spark stores data in memory utilizing Resilient Distributed Datasets (RDDs). Clients can utilize the iterative activity to peruse information from the disc and compose it to RDDs to assemble a task. Alternatively, they can use intuitive mode to run numerous questions on a similar subset of data [26]. The Spark Streaming is a decent stream-preparing alternative [27]. Figure 2 throws light on Apache Spark and its relation to the BigDL library for deep learning.

Deep learning is a subset of state-of-the-art artificial intelligence calculations, frequently known as deep organized learning. It is subject to mathematical action procedures and Artificial Neural Networks [28]. This methodology extricates and changes attributes by using various secret layers. Each layer gets the yield of the first one as info, acknowledges contributions to be prepared, and means to deliver the estimation’s outcome [29].

Deep-learning approaches empower machines to learn without the requirement for programming. Automatic speech acknowledgment, natural language handling, medical prescription turn of events and toxicology, client relationship management, recommendation frameworks, and bioinformatics are only a couple of the issues that deep learning has been utilized to address [30]. Spark with deep understanding is also used in [31].

The CNN (Convolutional Neural Network) is an artificial neural network powered by an animal’s visual cortex [32]. A deep learning algorithm can order input pictures, for example, chest X-ray pictures as tainted or not through COVID-19 [33]. The CNN will separate features, making grouping quicker and more exact. The CNN’s four fundamental layers are the Convolution, Non-Linearity (ReLU), Pooling, and Classification layers [34,35].

Transfer Learning (TL) is a procedure for moving acquired data to an original dataset to all the more likely process another destination dataset. TL happens when a model is reused as the beginning stage for another undertaking. One of the critical spaces of utilization for TL [36] is picture recognition. Rather than old-style learning, which is secluded and centred around singular tasks, transfer learning permits us to apply the knowledge on recently prepared models to foster new models and even tackle difficulties, for example, having less information for the new assignment.

The main intention of this investigation is to make a Deep Transfer Learning (DTL) structure utilizing Convolutional Neural Network (CNN) with the Apache Spark big data platform, in light of pre-arranged models InceptionV3, ResNet50, and VGG19. This work was inspired by deep-learning work in [37,38,39,40].

Our contribution is significant, which has been described in bullet points:

- We propose a novel approach with logistic regression for features extraction and CNN-based architectures of pre-trained VGG19, InceptionV3, and ResNet50 to detect COVID-19 from chest X-rays;
- To the best of our knowledge, our architectures with state-of-art architectures are effective and accurate after using the big-data framework, the Apache Spark in the pipeline;
- We appraise our architectures, classifying 100% in cases of COVID-19 and healthy patient chest X-ray images.
- Finally, our datasets are considerable, consisting of 1063 images for a 3-class classifier and 708 total images for a 2-class classifier.

The following is how the rest of the article is organized. Section 2 is about related work. Materials and methodology are explained in Section 3. The approach and outcomes of the experiments are detailed in full in Section 4. Section 5 consists of a prolonged discussion. Finally, Section 5 brings the conclusion.

## 2. Related Work

Deep learning approaches are mostly without big data. But some approaches to deep learning have been used without Spark. Their advantages and disadvantages have been discussed here. The study [41] used the CoroDet model has two class-based classifications of COVID-19 positive and negative patients. There are three types of classifiers: two-class (COVID and healthy patients), three-class (COVID positive patients, healthy person, and non-COVID pneumonia infected person), and four-class (COVID positive patients, healthy person, non-COVID pneumonia infected person, and non-COVID bacterial pneumonia). For low-quality X-ray pictures, the model performed poorly. According to the author, it has the most extensive dataset, and it outsmarted all previously used models in terms of accuracy. In the future, authors will want to use a larger dataset.

This study presents an automated, low-cost, rapid, and high-performance method for detecting COVID-19 disease. In-depth features from CT scan pictures are recuperated utilizing a CNN with a pre-trained CNN-based DenseNet201 design and transfer learning in [42]. The execution was evaluated using an Extreme Learning Machine (ELM) classifier. It outperformed the pre-trained models. The utilization of different ELM classifier initiation functions is dependent on deep features. In the future, authors will want to make web and mobile applications for the help of doctors using this technology.

A deep-learning algorithm named “COVIDetection-Net” was proposed in [43] to recognize coronavirus from chest radiography pictures. The proposed system utilizes the ShuffleNet and SqueezeNet designs for deeply learned attributes, just as it uses Multiclass Support Vector Machines (MSVM) for recognition and characterization.

The algorithm in [44] can group the information into three classifications: corona-positive patients, other pneumonia-infected patients, and no contaminations. Three particular learning algorithms are utilized to make the model: CNN, VGG-16, and ResNet-50. The chest X-ray is from Kaggle’s library. VGG16 beat CNN and ResNet-50 in each of the three learning algorithms to assess the model’s performance. Just two current COVID-19 mechanized screening strategies are contrasted with the version of the suggested TLCoV model. Data augmentation is additionally utilized. As a result of its predominant dynamic directing technique, the creator’s model uses the Capsule Network.

Three deep CNN models, AlexNet, GoogleNet, and ResNet, were pre-trained utilizing transfer learning to establish model parameters [45]. Softmax was used as the fully connected layer’s classification technique. Relative majority voting was used to obtain the ensemble classifier EDL-COVID. According to the findings, the ensemble model outperformed the component classifier in terms of overall classification performance.

The creators of [46] made two sickness diagnosis algorithms: a deep neural network (DNN) because of picture fractal properties and a CNN strategy that examines lung pictures straightforwardly. The segmentation method is also used to locate sick tissue through the CNN model.

The authors have presented a quick detection method based on X-ray image processing that they believe would benefit society. In [47], the authors proposed using X-ray scans to discover COVID-19 patients using the nCOVnet algorithm. In AI, they employed deep learning. In under 5 s, the proposed model could identify a COVID-19 positive patient. With the bit of data accessible, the creators could accomplish a valid positive rate of 97.62 percent. Many of the previous studies that claimed accuracy of up to 98 percent did not account for the possibility of data leaking, which the authors addressed while training nCOVnet, resulting in impartial results.

In [48], machine learning models were used to predict COVID-19 spread. Similarly, big data analytics is being used extensively to detect other diseases and drugs, as mentioned in [49,50,51,52].

## 3. Materials and Methods

This section presents the dataset description and methods. Section 3.1 belongs to the description of the dataset. Finally, the proposed big-data approach is explained through pre-trained CNN models ResNet50, InceptionV3, and VGG19 models.

### 3.1. Coronavirus X-ray Images Dataset

Chest radiograph or chest X-ray pictures were used in this investigation through two datasets. These images were taken from the Kaggle repository and came from two datasets: “Coronavirus chest x-ray images” [53] and “Chest X-Ray images (Pneumonia)” [54].

#### 3.1.1. Two-Class Classifier Dataset Description

In a two-class classifier, 708 X-ray images are used altogether, separated into two classifications: 354 COVID-19 infected patients’ X-ray images and 354 normal X-ray images. A dataset of 354 typical and 354 COVID-19 patients was assembled utilizing front-facing projections of chest X-ray pictures. The images used for testing were unknown for models. COVID-19 infected patients’ X-ray images were labeled with 1, while normal X-ray images were marked with 0. Table 1 describes the dataset sample of two classes.

#### 3.1.2. Three-Class Classifier Dataset Description

In the three-class classifier, there were 1063 images from the two databases mentioned above. It had three classifications: 354 pictures for COVID-19 patients, 355 for pneumonia-infected patients, and 354 for healthy individuals. COVID-19 infected patients’ X-ray images were labeled with 1. Normal X-ray images were tagged with 0. At the same time, pneumonia-infected chest X-ray images were marked with 2. Table 2 describes the number of samples of the three classes.

**Figure 3:**
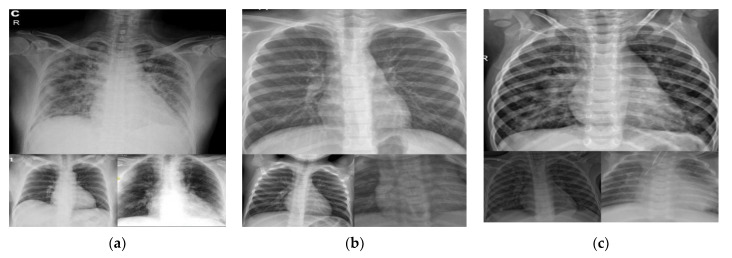
Sample X-ray images (**a**) COVID-19 lung X-ray images; (**b**) Healthy chest X-ray images; (**c**) Pneumonia chest X-ray images.

Figure 3a–c are the samples of X-rays of COVID-19, healthy, and pneumonia chest images, respectively.

### 3.2. Our Approach

**Figure 4:**
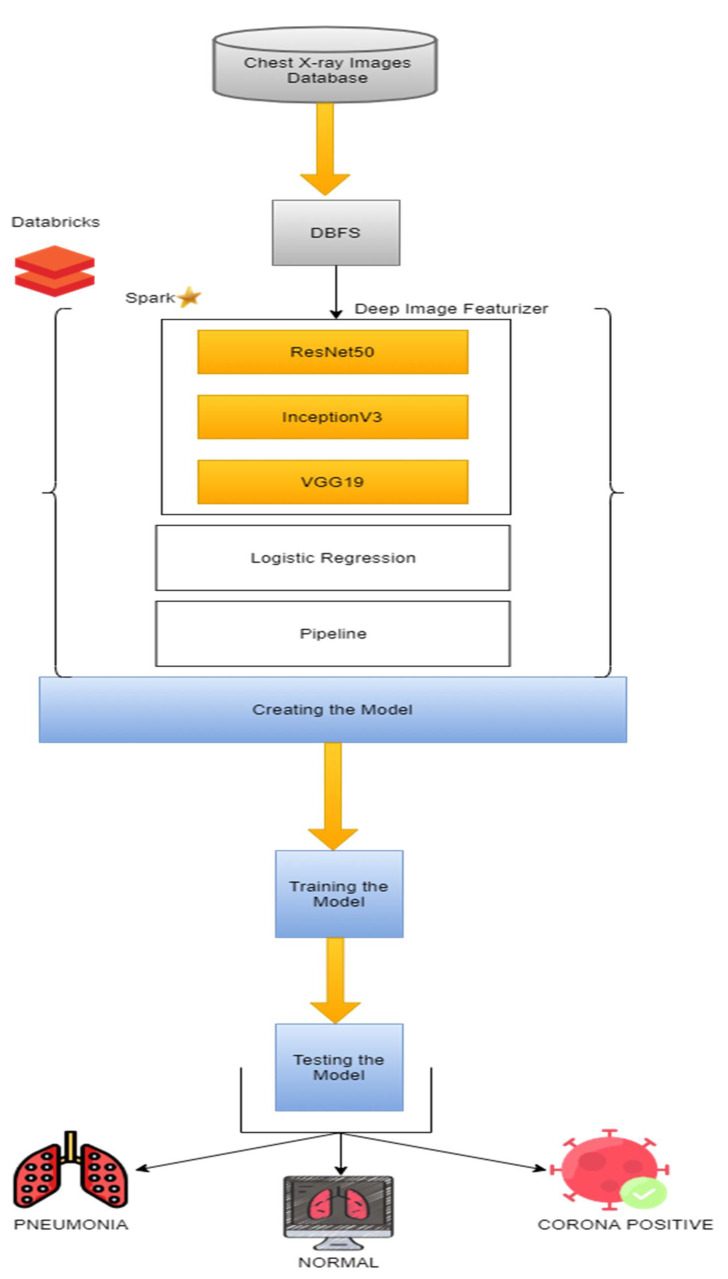
The working model incorporates deep-transfer learning with the Apache Spark architecture InceptionV3, Residual Net (ResNet50), and Visual Geometry group (VGG19) on Data Bricks File System(DBFS).

The transfer learning pipelines were used on Apache Spark in this exploration. The pre-arranged CNN architecture InceptionV3, Residual Net(ResNet50), and Visual Geometry group (VGG19) models were utilized along with logistic regression to sort out the chest X-ray pictures on Data Bricks File System(DBFS) of three classes. Figure 4 shows the architecture of our approach.

For deep learning at a more profound level, Databricks Runtime ML incorporates the Pipelines library.The framework architecture of the Pipelines is a deep-learning open-source project. It is a significant level system that utilizes Apache Spark through machine lerning and deep learning as well [55,56,57].

Logistic regression is a machine-learning-based statistical method for dissecting autonomous highlights that characterize a result. This model used three types of pictures (COVID-19 patients, pneumonia patients, and non-infected healthy persons)[58]. On ImageNet, there are three pre-trained designs: InceptionV3, ResNet50, and VGG19 models with loads.

The 3rd cycle of Google’s Inception CNN network model is Origin V3 [59] a deep neural network for image analysis and object recognition based on TensorFlow. A Residual Network with 50 layers is known as ResNet50 [60]. ResNet is a subclass of CNN that is most often utilized in picture acknowledgement and grouping. At the same time, VGG19 evolved from the VGG model, which is also the CNN evolution.

The InceptionV3 has been created for the identification of images. The Inception network was an essential milestone in the development of CNN classifiers. Before its inception, most popular CNNs stacked convolution layers deeper and deeper, hoping to get better performance. The Inception network, on the other hand, was complex [61]. Its constant evolution leads to the creation of several versions of the network. InceptionV3 incorporated needed upgrades stated for InceptionV2 [62].ResNet, short for Residual Networks, is a classic neural network used as a backbone for many computer vision tasks [63]. ResNet50 is a combination of 48 convolution layers along with one MaxPool and one Average Pool layer [64]. The main innovation of ResNet is the skip connection [65]. The VGG19 is the modified form of the VGG [66]. All three models were improved from conventional CNN models. It can be easily used along with transfer learning for image classification. It has 16 layers. This model will be smartly devoted to specialists’ help settling on better COVID choices and urging medical imaging experts to receive new methodologies as an asymptomatic instrument.

## 4. Experiment and Results

### 4.1. Experiment Setup

The Databricks Workspace was utilized for testing [67]. It is an analytics stage based on Apache Spark. It is a cloud-based platform similar to the Google Colab cloud environment [68]. Databricks is a synergistic platform that permits clients to concentrate the entirety of their logical tasks and oversee machine-learning models over as long as they can remember the cycle. A group has been developed in this platform to run the model according to the assortment of guidelines.

The Databricks File System was utilized to save the chest X-rays pictures (DBFS). It is a dispersed record framework that allows storing of information for inquiries inside Databricks and makes it reachable across clusters. A cluster was created using Apache Spark.

In a two-class classifier, meaning it has two paths to store. First is the path of the typical images, and the second path is chest X-rays about COVID-19. A three-class classifier has three paths to store. The first two paths are similar, and the third path is about pneumonia chest X-ray images. The dataset was used 80% for training and 20% for testing. Therefore, the model was trained on 566 X-ray images, and 142 images were used for testing. Moreover, 74 were normal X-ray images and 68 were COVID-19 infected patients’ X-ray images.

In the case of the three-class classifier, the dataset was used 80% for training and 20% for testing. Therefore, the model was trained on 856 X-ray images, and 207 images were used for testing. The images used for testing were unknown for models. Moreover, testing data was divided. There were 74 normal X-ray images, 66 COVID-19 infected patients’ X-ray images, and 67 pneumonia infected patients’ X-ray images.

### 4.2. Evaluation Metrics

We calculated our results using mean accuracy, precision, recall, confusion matrix, area under curves, and training losses.

The deep-learning approach is applied through logistic regression to extract features. In this examination, three CNN-based models were prepared and tried on chest X-ray pictures to identify COVID-19 or pneumonia-infected people utilizing Apache Spark. The deep-learning pipelines were used to stack photos into a Spark DataFrame, and views influenced by COVID-19 and ordinary pictures were named with the qualities “1” and “0” individually. Infected chest X-ray images were labelled with the quality “2” in the three-class classifier pneumonia.

The idea of a featurizer in deep learning pipelines empowers quick transfer learning on an Apache Spark cluster. A DeepImageFeaturizer was utilized, and the InceptionV3, ResNet50, and VGG19 models were used for this examination. Four measurements were used to assess the model’s exhibition in this investigation: accuracy, weighted recall, and weighted precision. The primary exhibition metric that was analyzed was accuracy. It alludes to how close the estimations are to a foreordained value.

The two-class classifier model performed with 100 percent accuracy with InceptionV3, ResNet50, and VGG19. The precision and recall also showed 100 percent results. This model correctly predicted samples for confirmation. In terms of the three-class classifier, InceptionV3, ResNet50, and VGG19 were 97, 98.55, and 98.55 percent, respectively. Table 3 shows the performance comparison of InceptionV3, ResNet50, and VGG19 for both binary and three-class classifiers. Table 3 shows the result of three models with two and three classes.

**Figure 5:**
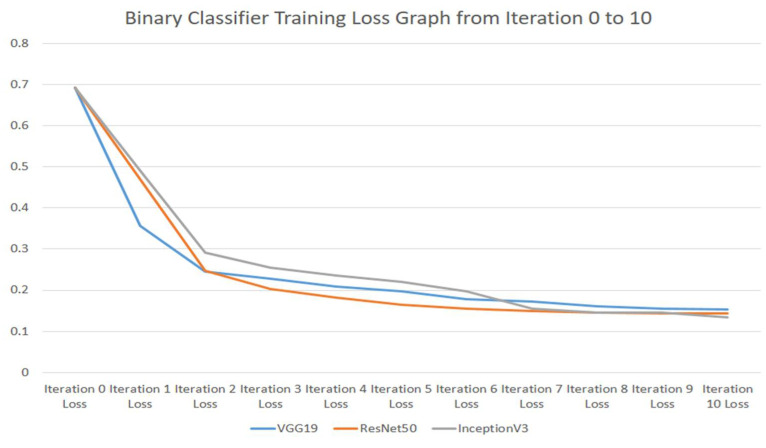
Binary classifier training-loss graph from iteration 0 to 10 for VGG19, ResNet50, and InceptionV3 Models.

The binary classifier training-loss graph from iteration 0 to 10 for VGG19, ResNet50, and InceptionV3 is shown in Figure 5.

**Figure 6:**
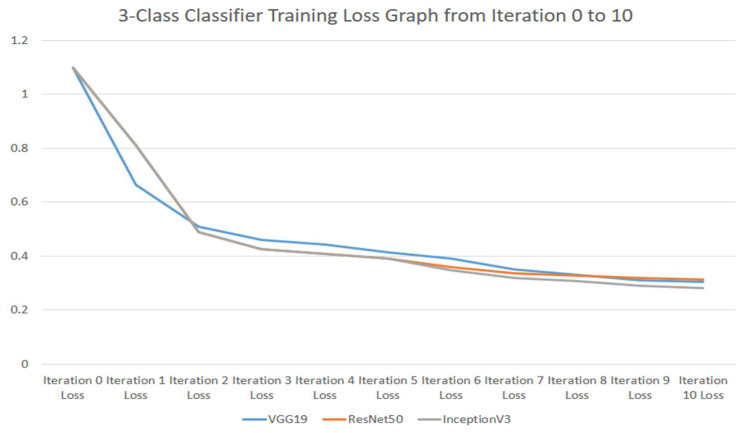
Three-class classifier training-loss graph from iteration 0 to 10 for VGG19, ResNet50, and InceptionV3 models.

The three-class classifier training-loss graph from iteration 0 to 10 for VGG19, ResNet50, and InceptionV3 is shown in Figure 6.

## 5. Discussion

**Figure 7:**
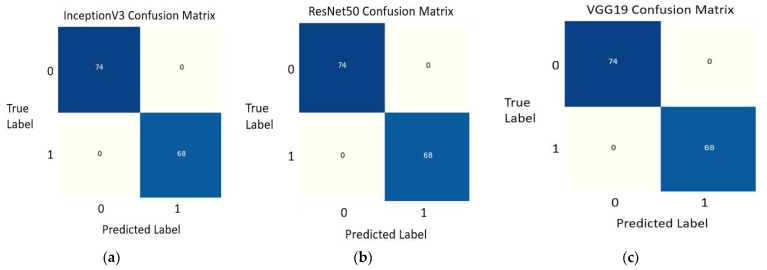
Two-class classifier confusion matrix; (**a**) Confusion matrix of Inception V3 model; (**b**) Confusion matrix of ResNet50 model; (**c**) Confusion matrix of VGG19 model.

In this work, an architecture for detecting COVID-19 in chest X-ray pictures was proposed based on deep transfer learning through the Apache Spark framework. According to the exploratory findings, our model attained an accuracy of 100% for InceptionV3, the ResNet50 Model, and VGG19. All of the most recent indices have confirmed 100% correct results. For the three-class classifier, the accuracy obtained for InceptionV3, ResNet50, and VGG19 was 97%, 98.55%, and 98.55%, respectively. The confusion matrices for two-class classifiers obtained for Inception V3, ResNet50, and VGG19 models are shown in Figure 7.

These confusion matrices show that 74 values were predicted correctly. Therefore, 74 values are True Positive (TP). It means that these X-ray images of COVID-19 patients were detected correctly. Similarly, 68 normal photos are not affected with COVID-19 and were also classified and detected correctly. This category lies in True Negative (TN). At the same time, no value lies in False Positive (FP) and False Negative (FN). The area under the curve is also 1.

**Figure 8:**
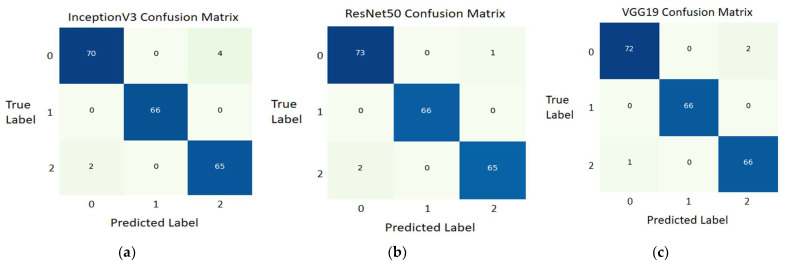
Three-class classifier confusion matrix; (**a**) Confusion matrix of InceptionV3 model; (**b**) Confusion matrix of ResNet50 model; (**c**) Confusion matrix of VGG19 model.

The confusion matrices for the three-class classifier obtained for Inception V3, ResNet50, and VGG19 models are shown in Figure 8.

The confusion matrix of InceptionV3 shows that 70 typical images’ values were predicted correctly, while four normal images were not predicted correctly. Similarly, 66 COVID-19 X-ray images and 65 pneumonia X-ray images were correctly predicted. Two pneumonia X-ray images were not predicted correctly.

The confusion matrix of ResNet50 shows that 73 normal images’ values were predicted correctly, while 1 normal image was not predicted correctly. Similarly, 66 COVID-19 X-ray images and 65 pneumonia X-ray images were predicted correctly. The two pneumonia X-ray images were not predicted correctly.

The confusion matrix of VGG19 shows that 72 normal images values were predicted correctly, while 2 normal images were not predicted correctly. Similarly, 66 COVID-19 X-ray images and 66 pneumonia X-ray images were predicted correctly. One pneumonia X-ray image was not predicted correctly.

**Figure 9:**
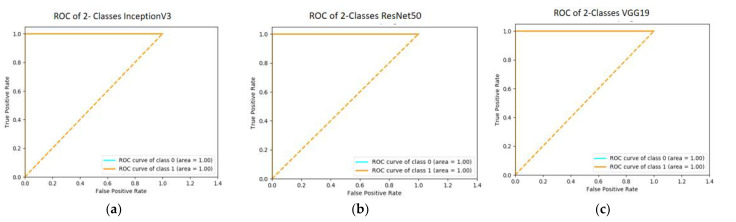

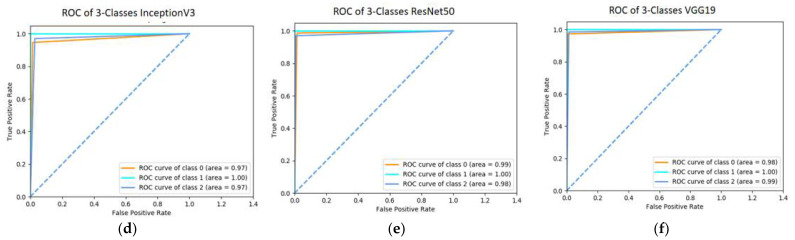
ROC AUC for two and three classes: (**a**) AUC two-class classifier InceptionV3 model; (**b**) AUC two-class classifier ResNet50 model; (**c**) AUC of two-class classifier VGG19 model; (**d**) AUC of three-class classifier InceptionV3 model; (**e**) AUC of three classifier ResNet50 model; (**f**) AUC of three-class classifier VGG19 model.

Figure 9 shows the binary-class classifier area under curve ROC plot and the 3-class classifier area under curve ROC plot for InceptionV3, ResNet50 and VGG19.

The Apache Spark system, joined with the deep-transfer learning strategy, created excellent execution, proficient examination, and progressed discoveries. Because of the combination of these three methodologies, our model can undoubtedly distinguish between corona-affected images and healthy chest X-ray images. We also compared our result with previous related studies in Table 4

The table, as mentioned above, compared the proposed model with some other similar works. This proposed model performed better as compared to other models in terms of performance and innovation. 

Aside from the excellent result, however, there are some limitations of our study. Firstly, our dataset is biased in the case of two classes. Secondly, if the noise in data increased, then the performance would also be decreased. Thirdly, our model is significantly slower due to an operation of logistic regression and transfer learning of various CNN architectures in the pipeline. Due to several layers, the training process takes a lot of time if the computer does not have a good GPU. Fourthly, the decisions of multiple radiologists are not considered in the final prediction. Lastly, our architectures have excellent performance while classifying images that are very similar to the dataset. However, if the images contain tilt or rotation, our architectures usually have difficulty organizing the image.

## 6. Conclusions

COVID-19 is a highly hazardous virus. World governments have adopted various methods to halt the spread of the virus, depending on their resources. Several countries expect new waves of this virus due to new deadly virus variants, and some impose lockdowns to control the spread. Countries are hastening their vaccination process according to their resources and availability of vaccine supply. Quick identification of positive corona patients and their isolation is effective in reducing spread, according to WHO. Because of this fact, a deep transfer-learning approach with Apache Spark architecture was created to recognize the coronavirus in chest X-ray images. Our image classification system relied on deep-learning pipelines and logistic regression, and three CNN-based models were used in this study, namely InceptionV3, ResNet50, and VGG19. Two databases from the Kaggle repository were utilized to create and test the model. A binary-class classifier and a three-class classifier were proposed in our architecture.

Databricks workspace has been used as an enormous data analytics platform to process X-ray images, and Apache Spark’s framework played a significant role in this process. In this investigation, weighted precision, weighted recall, and accuracy were examined as execution measurements for deep transfer learning. For InceptionV3, ResNet50, and VGG19, the outcomes were phenomenal. These three models named InceptionV3, ResNet50, and VGG19 gave 100% accuracy for binary-class classification. All performance measurements proved that these three models are predicting 100% correctly. InceptionV3, ResNet50, and VGG19 gave 97%, 98.55%, and 98.55% accuracy, respectively, when the 3-classes were classified. It has been ensured that architectural design can accurately detect coronavirus infection in chest X-ray images based on the outcomes generated by our model.

These findings may persuade health professionals worldwide to utilize these cutting-edge strategies to combat the coronavirus pandemic. A model will be developed for sudden spikes in demand for gigantic PC clusters utilizing the blend of its last two advancements, which makes the workplace intriguing. This model will make coronavirus detection simpler, quicker, and more affordable.

In future work, the integration of TensorFlow into Apache Spark will foster a new model for detecting coronavirus in chest X-rays, magnetic resonance imaging, and CT scans on a 4-class classifier.

## Figures and Tables

**Table 1:** Numerical description of the prepared dataset for a binary classifier.

The Classes	Number of Images
COVID-19	354
Normal	354

**Table 2:** Numerical description of the prepared dataset for three-class classifier.

The Classes	Number of Images
COVID-19	354
Normal	354
Pneumonia	355

**Table 3:** Overall performance comparison of 3-class and binary classifier InceptionV3, ResNet50, and VGG19 models.

Model	Classes	Mean Accuracy	Precision	Recall	Mean AUC
Inception V3	COVID-19, Normal	1	1	1	1
	COVID-19, Normal, Pneumonia	97.10%	0.9713	0.9710	0.9784
ResNet50	COVID-19, Normal	1	1	1	1
	COVID-19, Normal, Pneumonia	98.55%	0.9855	0.9855	0.9890
VGG19	COVID-19, Normal	1	1	1	1
	COVID-19, Normal, Pneumonia	98.55%	0.9855	0.9855	0.9893

**Table 4:** Comparing the state of the artworks with our proposed model.

Author, Year	Architecture	2 Class	3 Class	4 Class
Hussain et al., 2021 [51]	Novel CNN Model CoroDet	99.1%	94.2%	91.2%
M. Turkoglu, 2021 [52]	ELM and Deep Neural Network	-	98.36%	-
Das et al., 2021 [54]	CNN, VGG-16 ad ResNet-50	-	VGG = 97.67% ResNet-50 = 96.41% CNN = 93.67%	-
Zhou et al., 2021 [55]	AlexNet, GoogleNet, ResNet and SoftMax for Classification	-	GoogleNet = 98.25% ResNet = 98.56% SoftMax = 98.56% The ensemble model outperformed the component classifier.	-
Hassantabar et al., 2020 [56]	Deep Neural Network (DNN) and Convolutional Neural network (CNN)	CNN = 93.2% DNN = 83.4%	-	-
Panwar et al., 2020 [57]	Deep learning neural network model using nCOVnet algorithm	97.97%	-	-
Proposed Model	InceptionV3, ResNet50, VGG19	Inception V3 = 100% ResNet50 = 100% VGG19 = 100%	Inception V3 = 97% ResNet50 = 98.55% VGG19 = 98.55%	-

## Data Availability

This dataset available online and anyone can be used.
